# Managing High Cardiac Output Failure in a Patient With Hereditary Hemorrhagic Telangiectasias During Pregnancy

**DOI:** 10.1016/j.jaccas.2023.102096

**Published:** 2023-12-06

**Authors:** Erica Lynn Smith, Robert Egerman, Arwa Saidi, Ki Park, Ali Ataya

**Affiliations:** a Department of Obstetrics and Gynecology, University of Florida, Gainesville, Florida, USA; b Congenital Heart Center, University of Florida, Gainesville, Florida, USA; c Division of Cardiology, University of Florida, Gainesville, Florida, USA; d Division of Pulmonary, Critical Care, and Sleep Medicine, University of Florida, Gainesville, Florida, USA

**Keywords:** arteriovenous malformation, bevacizumab, hereditary hemorrhagic telangiectasias, high cardiac output failure, liver hemangioma, pregnancy

## Abstract

Hemorrhagic telangiectasias is a rare genetic vascular disorder that may complicate pregnancy. We report a case of a pregnant hemorrhagic telangiectasias patient with innumerable hepatic arteriovenous malformations that developed high output cardiac failure necessitating delivery. Postpartum, the patient was treated with bevacizumab that resulted in clinical improvement.

## Central Illustration

**Figure:**
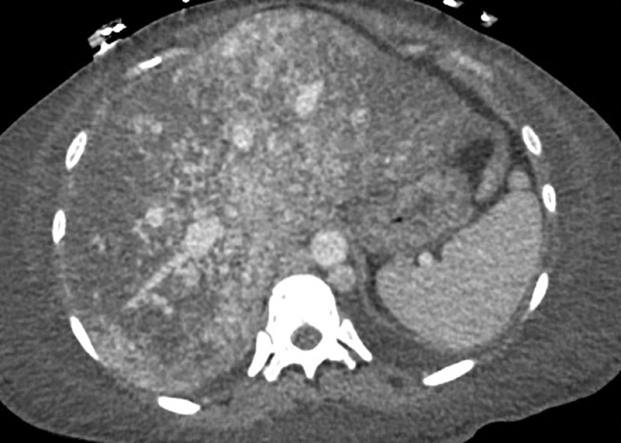

## History of Presentation

A 39-year-old primigravida female patient with known hereditary hemorrhagic telangiectasia (HHT) presented at 32 weeks’ gestation with progressive lower extremity swelling, shortness of breath, and epistaxis. The patient’s prenatal course was complicated by increasing episodes and severity of epistaxis requiring packed red blood cell transfusions during her third trimester despite topical treatments. On clinical examination, the patient was tachycardic with evidence of +3 pitting edema to the abdomen, jugular venous distention, and a new oxygen requirement.

**Learning Objectives**

• To recognize the signs of high CO failure in the setting of pregnancy.
• To understand how liver AVMs in HHT may result in high CO failure.
• To understand the treatment options for high CO failure in HHT.

## Past Medical History

Her medical history was only relevant for HHT with autosomal dominant pathogenic *ACVRL1* mutation (c.1121 G>A), complicated by cerebral vascular malformation during childhood that was coiled, mild epistaxis, and liver arteriovenous malformations (AVMs).

Prior to pregnancy, her HHT course had been stable with mild and infrequent nosebleeds that never requiring iron or blood transfusions or any other medical therapies.

## Differential Diagnosis

Differential diagnoses include preeclampsia, physiologic high cardiac output (CO) state during pregnancy, pulmonary hypertension, and high CO heart failure in setting of HHT.

## Investigations

**Figure 1:**
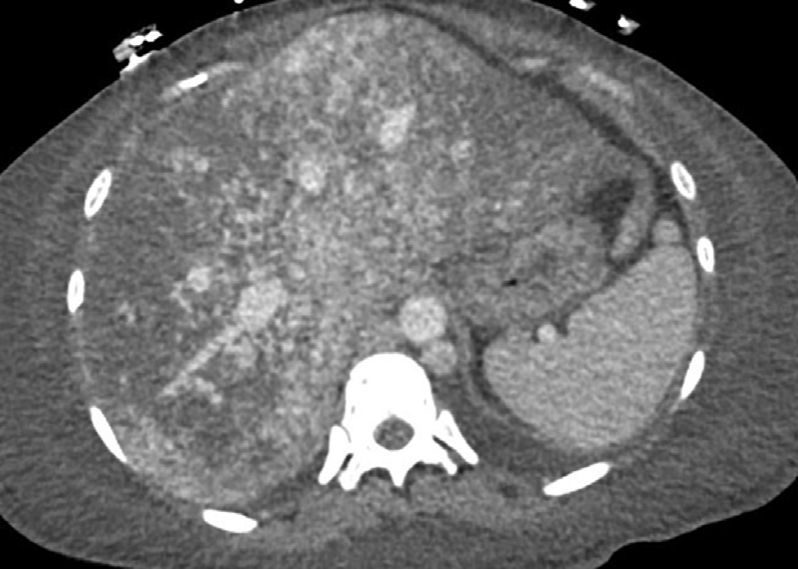
3-Phase Liver Computed Tomography Innumerable liver venous malformations visualized with computed tomography.

**Figure 2:**
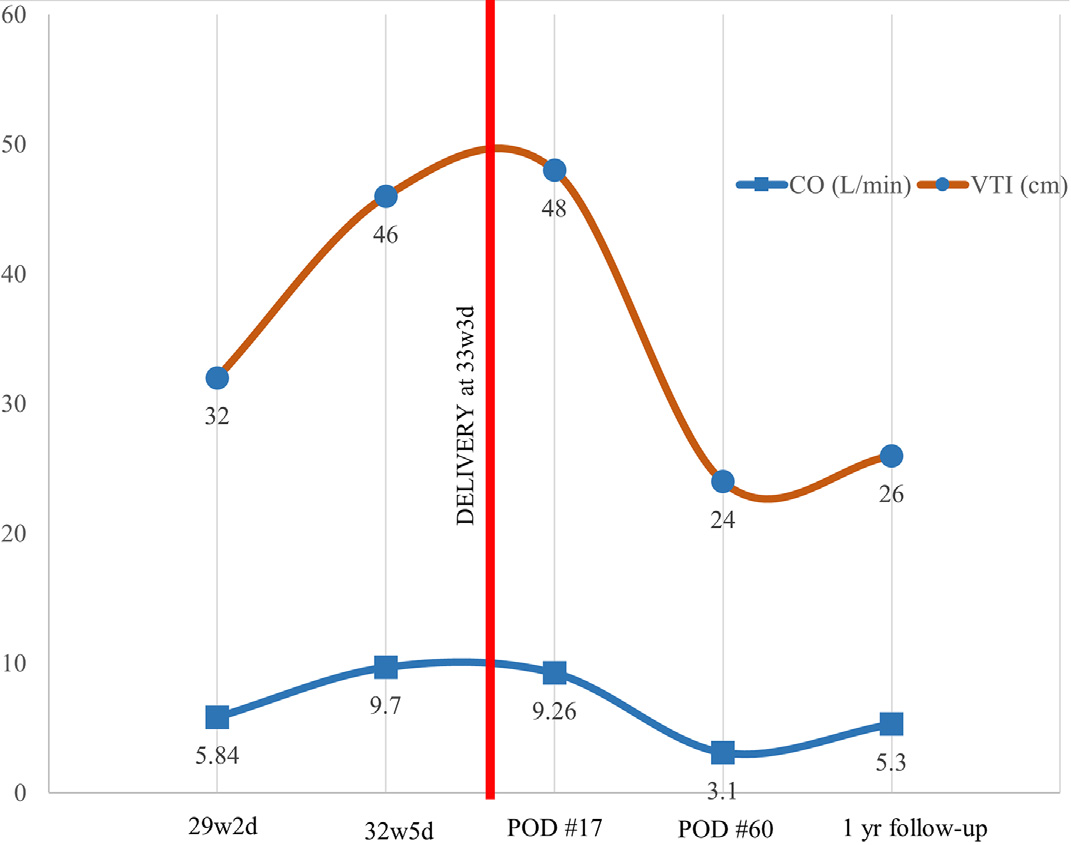
Results of Transthoracic Echocardiogram Across Pregnancy and Postpartum Demonstrating change in cardiac output (CO) and velocity time interval (VTI) throughout the course of care. POD = post operative day.

A transthoracic echocardiogram exhibited a hyperdynamic state (Video 1) with ejection fraction of 70% and increased estimated right ventricular systolic pressure at 45 mm Hg. The estimated CO was 9.7 L/min with no evidence of shunting. A 3-phase liver computed tomography revealed hepatomegaly with innumerable vascular malformations (Figure 1) with duplication of the inferior vena cava with azygous continuation of the inferior vena cava. Her last prior echo at 29 weeks’ gestation was normal with estimated CO of 5.8 L/min (Figure 2).

## Management

Due to progression of heart failure symptoms despite aggressive diuresis, after multidisciplinary discussion among the HHT Center of Excellence team and the patient, she underwent a cesarean section with bilateral tubal ligation at 33 weeks 3 days of gestation. Following delivery, epistaxis continued, requiring additional transfusions despite nasal cauterization and intranasal bevacizumab injections. She underwent a right heart catheterization 3 weeks after delivery that showed postcapillary pulmonary hypertension with high CO state with a right atrial pressure of 17 mm Hg, mean pulmonary arterial pressure of 30 mm Hg, pulmonary capillary wedge pressure of 27 mm Hg, and a CO and index (via thermodilution) of 7.4 L/min and 4.7 L/min/m^2^, respectively. Given her symptoms of high CO failure in the setting of liver AVMs and the worsening epistaxis, systemic bevacizumab 5 mg/kg was administered every 2 weeks for a total of 6 doses followed by maintenance dosing every 6 months. Patient did not breastfeed because she was on therapy.

## Discussion

CO in pregnancy normally increases 45% by 24 weeks’ gestation. In early pregnancy, CO is mediated by an increase in stroke volume accompanied by an increase in preload from expanding plasma volume and a decrease in afterload through less vascular resistance. Later in gestation, the increased CO is attributable to increasing heart rate. In the first half of pregnancy, stroke volume gradually increases, and then it stabilizes at approximately 24 weeks, remaining constant or decreasing later in pregnancy.^1^

There are many mechanisms for high CO failure that include increased flow through the venous circulation. This lack of resistance results in increased oxygen consumption and low systemic vascular resistance. AVMs can lead to this high venous flow circulation. AVMs in pregnancy have been demonstrated to result in high CO failure if >20% of the CO is shunted through the AVM. The combination of pregnancy and AVM has been proposed to result in a 150% increase in CO.^2^ There are case reports describing high CO failure when fistulas have formed in multiple organ systems including hepatic, pulmonary, mesenteric, renal, and congenital port wine staining. HHT and high CO failure have been described in the setting of hepatic AVMs.^3^, ^4^, ^5^, ^6^

Use of bevacizumab has been helpful for treatment of some of the manifestations of HHT.^7^ Bevacizumab is a recombinant humanized monoclonal immunoglobulin antibody blocking VEGFA. The drug is teratogenic because animal models demonstrate birth anomalies, low body weight, and fetal deaths in the rats, and systemic levels of bevacizumab are reported in intravitreal injections of primates. Use of bevacizumab during pregnancy is limited to only case reports with most including unintentional first trimester exposures.^8^ There was hesitancy to use bevacizumab in the postpartum period due to the patient’s desire to breastfeed, but bevacizumab is a large molecule 149,000 Da in size that exceeds the 600-Da size limit for excretion into breast milk. No detectable bevacizumab has been demonstrated in breast milk in 1 study when it was given intravitreally.^9^

## Follow-up

Patient responded successfully to bevacizumab, and after the second induction dose, she experienced significant improvement in her symptoms and resolution of her high CO failure. Assessment of her response was based on clinical improvement of both high CO heart failure symptoms and epistaxis as well as changes in estimated CO state by echo. She did not undergo a follow-up right heart catheterization. After 2 infusions she started having significant improvement, but she was not back to baseline until after the induction phase of bevacizumab (6 infusions every 2 weeks, which is approximately 3 months). It is possible that changes in the high volume state during pregnancy may have played a role in her improvement as well; however, she did not immediately improve after delivery until bevacizumab was initiated. She did not breastfeed after agreeing to start therapy. She continued maintenance therapy every 6 months with normalization of her echo and improvement in her epistaxis.

Neonatal genetic counseling and testing was completed and was found to be negative for the *ACVRL1* mutation.

## Conclusions

Patients with HHT can develop various serious complications during pregnancy that require a multidisciplinary team approach. In the setting of liver AVMs, the development of high CO failure is a serious complication that may necessitate early delivery. Systemic bevacizumab should be considered because it can reverse the high CO state and improve epistaxis in these patients.

All patients with known HHT considering pregnancy should be referred for pregnancy counseling with a maternal fetal medicine specialist, be seen at an HHT center of excellence, and undergo evaluation and updated screening, ideally prior to pregnancy. This evaluation should include bubble echo to evaluate for high CO state and presence of pulmonary AVMs and pulmonary hypertension. Brain imaging for cerebral vascular malformations, if not already completed as an adult, should be performed. Right heart catheterization should be offered only if indicated by echo. Contraceptives are not contraindicated as long as the patient is deemed stable and has no other comorbidities. Based on current evidence, bevacizumab should be avoided during pregnancy.

## Funding Support and Author Disclosures

The authors have reported that they have no relationships relevant to the contents of this paper to disclose.

## Appendix

Video 1 Transthoracic Echocardiogram: Parasternal Long-axis View Demonstrating Hyperdynamic Left Ventricular Function
